# The effects of a moderate physical activity intervention on physical fitness and cognition in healthy elderly with low levels of physical activity: a randomized controlled trial

**DOI:** 10.1186/s13195-022-01123-3

**Published:** 2023-01-11

**Authors:** Sara A. Galle, Jan Berend Deijen, Maarten V. Milders, Mathieu H. G. De Greef, Erik J. A. Scherder, Cornelia M. van Duijn, Madeleine L. Drent

**Affiliations:** 1 Department of Clinical, Neuro- and Developmental Psychology, Vrije Universiteit Amsterdam, Van Der Boechorststraat 7, 1081 BT Amsterdam, The Netherlands; 2 Department of Epidemiology, Erasmus Medical Center, PO Box 2040, 3000 CA Rotterdam, The Netherlands; 3 Hersencentrum Mental Health Institute, Marnixstraat 364, 1016 XW Amsterdam, The Netherlands; 4 Human Movement Sciences, University of Groningen, University Medical Center Groningen, PO Box 196, 9700 AD Groningen, The Netherlands; 5 Nuffield Department of Population Health, University of Oxford, Old Road Campus, Headington, Oxford, OX3 7LF UK; 6 Big Data Institute, Li Ka Shing Centre for Health Information and Discovery, Old Road Campus, Headington, Oxford, OX3 7LF UK; 7 Department of Internal Medicine, Endocrinology Section, Amsterdam University Medical Center, PO Box 7057, 1007 MB Amsterdam, The Netherlands

**Keywords:** Aging, Cognition, Physical activity, Physical fitness, Apolipoprotein E, Randomized controlled trial

## Abstract

**Background:**

Increasing physical activity is one of the most promising and challenging interventions to delay or prevent cognitive decline and dementia.

**Methods:**

We conducted a randomized controlled trial to assess the effects of a physical activity intervention, aimed at increasing step count, in elderly with low levels of physical activity on measures of strength, balance, aerobic capacity, and cognition. Participants were assigned to 9 months of exercise counseling or active control.

**Results:**

The intention-to-treat analyses show that the intervention, compared to control, increases the level of physical activity, but has no significant effect on physical fitness and cognition. Those who increased their physical activity with 35% or more show significant improvements in aerobic capacity, gait speed, verbal memory, executive functioning, and global cognition, compared to those who did not achieve a 35% increase.

**Limitations:**

The number of participants that achieved the intended improvement was lower than expected.

**Conclusion:**

Responder analyses suggest an improvement of physical fitness and cognition in those who achieved an increase in physical activity of at least 35%.

**Trial registration:**

The trial protocol is registered at the Dutch Trial Register NL5675, August 1, 2016.

## Background

The Lancet commission on dementia prevention, intervention, and care has indicated 12 modifiable risk factors for dementia and estimates that these factors account for 40% of all dementias worldwide that could potentially be prevented or delayed [1]. Physical activity is a promising intervention target to delay cognitive decline and dementia [1–3]. Yet, there is also substantial evidence of reverse causality [4, 5]. Subclinical neuropathological changes that proceed the diagnosis of dementia can gradually affect behavior, reducing physical activities as much as 9 years before the diagnosis of dementia [5]. Meta-analyses aggregating the effect of experimental trials report that physical activity interventions have modest positive effects on cognition [6–10]. Heterogeneity among these studies have led others to conclude the evidence is inconsistent or insufficient [11–14].

Important unanswered questions concern the parameters, mediators, and moderators of successful physical activity interventions. The most consistent positive results are observed in interventions of moderate intensity [8, 11, 15, 16], with a duration of at least 6 months [7, 11, 13, 17], aimed at people with low levels of physical activity [18, 19]. Intervention potential could be enhanced by targeting “at risk” individuals, defined by various health parameters including level of physical activity, genetic susceptibility, cardiovascular risk profile, and age.

Although physical activity is known to have an inverse association with several chronic diseases associated with cognitive impairment and dementia, such as cardiovascular disease, hypertension, diabetes mellitus, and obesity [20–31], it is unclear whether the temporary upregulation of the level of physical activity is enough to elicit the desired cardiovascular and neurocognitive changes. Observational data from epidemiological studies indicate that physical activity may take years to impact cardiovascular brain health [32, 33], and systematic reviews of RCTs fail to show consistent evidence of a relationship between physical fitness and cognitive performance [16, 34].

The relatively short-term improvements in cognitive functioning observed in intervention studies may be driven by mechanisms other than the improvement of physical fitness, like the promotion of cerebral angiogenesis and synaptic plasticity elicited by stimulation of neurotrophic factors such as brain-derived neurotrophic factor (BDNF), insulin growth factor 1 (IGF-I), and vascular endothelial growth factor (VEGF) [14, 16, 34–36].

Furthermore, observational studies investigating effect moderation by *APOE* genotype have indicated that higher levels of physical activity might help attenuating both cognitive deficits and risk for Alzheimer’s disease more so in carriers than in non-carriers of the ApoE-ε4 allele [32, 37–44].

Improving physical activity in those who fail to meet the public health recommendations for older adults of 150 min of moderate intensity aerobic activity per week [13] independently, requires a tailored approach. The present study is aimed at durable behavior modification using the COACH method. The COACH method is an individually tailored exercise counseling method based on the Motivational Interviewing Technique and goal-setting theory, both considered to be effective instruments for behavioral modification [45, 46]. Application of this method has proven feasible in various patient groups [47–53] and increased the physical activity of older adults with an average of 33% after 3 months and 41% after 12 months [50].

In the present study, we conducted a 9 month randomized trial to assess whether an increase in physical activity of 35% or more, generated by the COACH method, can lead to an improvement in physical fitness, and subsequent improvements in cognitive functioning, and well-being of healthy elderly with and without a genetic vulnerability for dementia, based on their *APOE* genotype. We targeted those with low levels of physical activity.

## Methods

### Design

The current study was a 9-month randomized controlled trial (RCT) with one intervention group and one control group. Participants could participate in the baseline measurement only (T0) or continue with participation in the trial, including follow-up measurements at 6 months (T1) and at 9 months (T2).

### Recruitment

Recruitment took place between September 1, 2015, and August 31, 2019. Participants were recruited among older adults (55 +) who previously participated in the Erasmus Rucphen Family Study (ERF) [54] and among the general population. Study information was spread via various media, including www.hersenonderzoek.nl. Four out of the 102 participants in the intervention study previously participated in the ERF-study.

### Participants

**Fig. 1:**
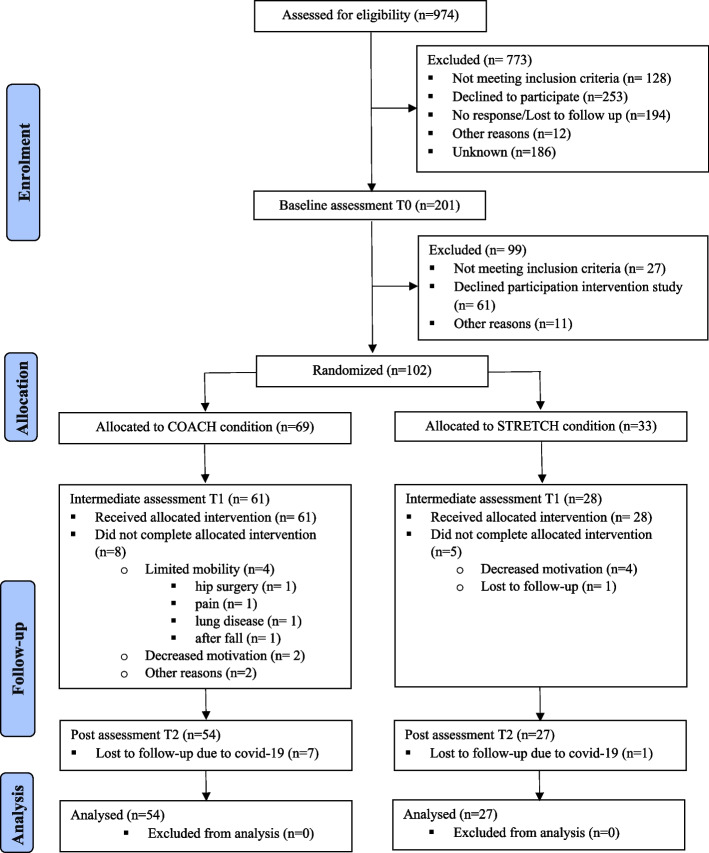
Flow-chart of participant selection

Eligibility criteria were as follows: aged ≥ 55 years, able to perform the Timed Up and Go Test (TUG) within 20 seconds or less [55], and a Mini-Mental Status Examination (MMSE) [56] score ≥ 25. Exclusion criteria were as follows: wheelchair-bound, cardiovascular problems that limit physical activity based on the Physical Activity Readiness Questionnaire (PARQ) [57], epilepsy, diagnosis of dementia or mild cognitive impairment, progressive or terminal disease, depression, history of alcoholism, severe visual or auditory problems, or difficulty with the Dutch language. Only those participants who reported low levels of physical activity at baseline were recruited for participation in the trial. Low levels of physical activity were defined as < 7000 steps a day on average. This number was calculated according to the Tudor-Locke translation of public health recommendations in minutes a day to steps a day for older adults [58]. Figure 1 displays a flow-chart of participant selection.

### Randomization and masking

Participants were assigned to the intervention or control condition by an independent researcher using a random number generator per block with an allocation of 2:1 in favor of the intervention. Although research assistants, exercise counselors, nor participants were informed of the existence of a control intervention, the trial could not be blinded as the difference in content and aims of the coaching and stretching sessions could not be concealed for participants, exercise counselors, or research assistants.

### Procedure

**Fig. 2:**
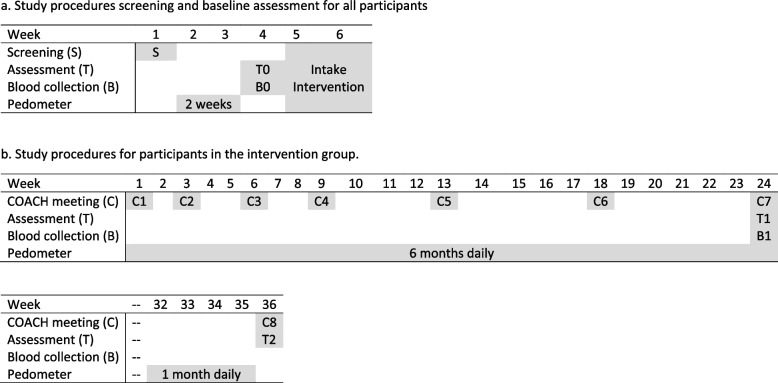
**a** Study procedures screening and baseline assessment for all participants. **b** Study procedures for participants in the intervention group. **c** Study procedures for participants in the control group

Eligibility screening was followed by the baseline assessment of all outcome measures including physical activity using a pedometer over two consecutive weeks (T0). Assessments of all outcomes were repeated at T1 and T2. Participant procedures are depicted in Fig. 2.

### Intervention

The intervention is an adapted version of the COACH protocol (www.coachmethode.nl), a pedometer-based exercise counseling strategy, aimed at enhancing low-to-moderate daily physical activity [51]. Physical activity goals are quantified in steps per day and pedometers are used as feedback instrument to monitor progress [59–63]. The original version of the COACH program spans a period of 3 to 4 months, including four to six coaching sessions. In the present study, the protocol was extended to seven coaching sessions, spanning 6 months and a follow-up session 9 months after the start of the intervention. In the coaching sessions of 45 minutes each, the counselor encourages participants to enhance daily physical activity such as walking, cycling, housekeeping, and gardening. The follow-up session (C8) is aimed at the evaluation and consolidation of the newly learned physical activity behavior. By stimulating low-to-moderate daily physical activity, the program is feasible for elderly people with varying levels of physical fitness. Adherence is encouraged by allowing participants to freely choose physical activity behaviors that match their preferences. The focus on step count facilitates the process of goal setting and goal monitoring.

The stimulation of daily physical activities by the exercise counselors was applied in four phases: (1) need to change; (2) benefits versus effort; (3) plan of action including specific, measurable, attainable, realistic, and time-bound (SMART) goals; (4) implementation plan. Participants were required to monitor their daily physical activity by means of a pedometer and record the total number of steps per day in a paper or online diary. For activities other than walking, participants were required to register duration, activity type, and intensity. The duration of these activities was converted into steps, according to the criteria of Tudor-Locke and Basset [58, 64], by using the adult cadence of 100 steps per minute. Based on previously described metabolic equivalents of task, the total number of steps assigned to vigorous activities was multiplied by factor 1.5 [65]. We converted non-ambulatory activity to steps as (1) locomotor activity constitutes the bulk of physical activity for adults [66] and (2) the use of step count facilitates the process of goal-setting and monitoring for the participants and (3) increases comparability across participants. Participants in the intervention group are instructed to use the pedometer during the 6 months of the intervention and at least 1 month before the last measurement 9 months after the start of the intervention. Adherence was determined by active participation in the coaching sessions and daily registration of step count. Table 1 presents a further specification of the content of the COACH sessions. Figure 2b specifies the study procedures per week for participants in the intervention and the control group.

**Table 1:** Specification of the content of the COACH sessions

Week	Phase	Content COACH session
1	Motivation to increase physical activity	- Introduction - Psychoeducation, WHO physical activity recommendations - Pedometer use - Current and historic pattern of physical activity: step average, activities, highs and lows - Expectations, personal preferences, struggles, resistance - Motivation, current stage of change - Mind map, decisional balance - Limitations and possibilities - Set long- and short-term SMART goals - Monitor step count with pedometer
3	Motivation to increase physical activity Goal setting	- Evaluate experiences, physical activity pattern, and goals - Encountered and anticipated barriers and setbacks - Examine conditions for change - Benefit versus effort - Set short-term SMART goals - Monitor step count with pedometer - Plan guarantees and alternatives
6	Goal setting	- Evaluate physical activity pattern and goals - Observe weekly fluctuations and external influences - Encountered and anticipated barriers and setbacks - Benefit versus effort - Set short-term SMART goals - Monitor step count with pedometer - Plan guarantees and alternatives
9	Goal setting	- Evaluate physical activity pattern and goals - Observe external and seasonal influences - Encountered and anticipated barriers and setbacks - Evaluate coping strategies - Benefit versus effort - Set short-term SMART goals - Monitor step count with pedometer - Plan guarantees and alternatives - Plan peak performance day
13	Shifting boundaries	- Evaluate physical activity pattern and goals - Evaluate peak performance day and personal physical activity norm - Observe external and seasonal influences - Encountered and anticipated barriers and setbacks - Coping strategies - Benefit versus effort - Monitor step count with pedometer - Set personal physical activity norm - Plan guarantees and alternatives
18	Consolidation	- Evaluate progress, adjust personal physical activity norm - Evaluate current repertoire of physical activities - Evaluate influence decreased coaching frequency - Encountered and anticipated barriers and setbacks - Coping strategies - Benefit versus effort - Monitor step count with pedometer - Plan guarantees and alternatives
24	Consolidation	- Evaluate coaching program, progress, and personal physical activity norm - Lessons learned - Evaluate influence decreased coaching frequency - Encountered and anticipated barriers and setbacks - Coping strategies - Benefit versus effort - Monitor step count with pedometer - Plan guarantees and alternatives
36	Consolidation	- Evaluate personal physical activity norm, COACH free period and coaching program - Moving forward and lessons learned

### Control condition

Participants in the control group received seven individually guided muscle stretching sessions, of 45 minutes each, scheduled over 6 months. Participants were offered a set of stretching exercises spanning the muscle groups of all body parts. During the sessions, participants were guided through the exercises of their choice and encouraged to perform these exercises outside of the session as desired. Figure 2c specifies the study procedures per week for participants in the control group. The follow-up session (S8) is aimed at the evaluation and consolidation of the newly learned stretching habit. Controls were requested to use the pedometer during 1 month before each follow-up measurement, as average daily step count is known to increase significantly in the first week of measurement when using unsealed pedometers and self-registration, due to reactivity [67]. The coaching sessions and the stretching and toning sessions were offered by a coach trained by the CBO (Centrum voor Beweging en Onderzoek Groningen).

### Baseline characteristics

Participants were asked about their health status, medication use, lifestyle habits, and sociodemographic factors. Education level was defined as low, middle, and high according to the Dutch classification of educational attainment [68]. Buccal cells were collected using a buccal swab and stored at − 20 °C. *APOE* genotyping of DNA samples was performed by the Department of Clinical Chemistry of the Amsterdam UMC. DNA was purified manually from buccal swabs [SOP 011036] and amplified through PCR [SOP 011909]. Cycle sequencing of amplified DNA samples [SOP 032929] (ABI prism Big Dye Terminator, Applied Biosystems by Life Technologies, Austin Texas, USA) and sequencing analysis were performed [SOP 011183] (Applied Biosystems 3130XL Genetic Analyzer Applied Biosystems by Life Technologies, Foster City, CA, USA). Determination of allele status occurred on basis of codons 112 and 158 of the *APOE* gene on chromosome 19. *APOE* genotypes were categorized into ApoE-ɛ4 carriers and non-carriers.

### Outcomes

#### Primary outcomes

##### Physical activity

Level of physical activity was expressed in average number of steps per day as assessed by a hip-worn pedometer. Participants were provided with one of the four following types of pedometers: the Omron walking style II, IV, and One 2.0 (Omron Healthcare Co. Ltd., Dallian, China), and the Yamax Digiwalker (SW-200, Yamasa Tokei Keiki Co. Ltd., Tokyo, Japan). All models use 3-axis technology to register ambulatory motion and have demonstrated good and comparable validity in free-living conditions [69]. Pedometers have a high construct validity [70] and are considered a valid and accessible option to assess physical activity. Pedometer measurements have been shown to correlate strongly (*r* = 0.86) with different accelerometers and with time in observed activity (*r* = 0.82) and moderately with different measures of energy expenditure (r = 0.68) [71]. A self-report questionnaire, the Physical Activity Scale for the Elderly (PASE) [72] was used to assess self-reported level of physical activity*.* The total PASE score is based upon the self-report of the frequency and duration of various physical activities performed in the past seven days, categorized according to their intensity. Higher scores indicate higher levels of physical activity.

##### Cognitive function

Cognitive function was assessed by neuropsychological tests summarized in composite scores for global cognition, executive functioning, learning, and verbal memory, based on theoretical considerations [73]. Higher scores indicate better cognitive ability. The global cognition score was created using the sum of the standardized scores of the 15 Word Test delayed recall (15-WT) [74], the Trail Making Test (TMT) B [75], the Stroop Color Word Test Interference score [76], the total scores on the Letter Fluency Test [77], and the Digit Span backward test [78, 79]. An executive functioning score was created using the sum of the standardized scores of the TMT AB-ratio, the Stroop Color Word Test Interference score, the total score on the Letter Fluency Test, and the Digit Span Test backward. Verbal episodic memory was assessed with the delayed recall condition of the 15-WT. A domain score learning was created for short-term memory performance using the Location Learning Test [80, 81] and the 15 Word Test. The ability to encode and imprint visual information was reflected in the total number of placement errors on the five trials of the Location Learning Test. The ability to encode and imprint verbal information was reflected in the number of correctly recalled words after five trials of the 15 Word Test. To create the learning index, both scores are standardized and the total number of placement errors on the LLT is inverted, so that for both measures higher scores indicate better imprinting ability.

##### Physical fitness

The Short Physical Performance Battery (SPPB) [82] was used to assess balance, lower body strength, and gait speed. The total balancing time in seconds is used as an indication of balance. Handgrip strength in kilograms was measured by the hydraulic JAMAR dynamometer [83–85]. A combined muscle strength score was created using the sum of the standardized scores of the SPPB sit-to-stand chair test and grip strength. The Six Minute Walk Test (6MWT) [86] is used to assess aerobic capacity [87]. Higher scores indicate better physical fitness. Six-meter walking speed in seconds is used as an indication of gait speed. Lower scores indicate better performance.

#### Secondary outcomes

##### Serum concentrations of the cardiovascular risk factor profile

Fasting blood samples are collected and centrifuged at 1800 g for 10 minutes, directly on site or after transport on the same day. Serum is frozen at − 80 °C until assessment. In all serum samples, total cholesterol, HDL-cholesterol, triglycerides, insulin, and IGF-I were assessed by the Department of Clinical Chemistry of the Amsterdam UMC.

##### Activities of daily living, frailty, and mental health

The Rand-36 mental health sub-scale [88] was used to assess psychological well-being. Higher scores indicate a more positive emotional state. Depressive symptoms were assessed with the Centre for Epidemiological Studies Depression Scale (CES-D) [89, 90]. Higher scores indicate more depressive symptoms. Limitations in the activities of daily living (ADL) were assessed using the Katz-15 scale [91], part of the TOPICS-MDS (www.topics-mds.eu). In this questionnaire, the need for assistance on 15 activities of daily living is inventoried. Higher scores indicate more ADL limitations.

A composite measure of frailty was calculated using the number of deficits in activities of daily living, social functioning, emotional well-being, and self-reported health on the TOPICS-MDS Higher index scores indicate more frailty.

### Statistical analyses

Sample size calculations are based on the assessment of the neuropsychological outcome measures using “G-Power 3.1.” Based on an expected effect size of *f* = 0*.*20 (equals *d* = 0.4), power = 0*.*80, alpha = *0.0*5, 2 groups, 3 measurements, and an estimation of the test–retest reliability = 0*.*6 [92–94], the required sample size for within-between interaction in a repeated measures analysis is (33*.*5) 34 subjects per group. We additionally calculated the power for unequal sample sizes by using the following equation $N=\frac{{(Z}_{\left(1-\propto \right)}+ {Z}_{(1-\beta )}{)}^{2}* {\sigma }^{2}*(\mathrm{r }+ 1) [1+T-1)\rho ]}{{v}^{2}T}$. Using an allocation ratio of *r* = 0.5, standard deviation of the outcome measurement $\sigma =1$, the number of follow-up measurements *T* = 2, correlation between measurements $\rho =0.6$, and the number of participants in the smallest group N = (33.5) 34 participants, the study is powered to detect a $v=0.47$ difference in mean value of the standardized outcome variable, corresponding to a medium effect size (*d* = 0.47).

The effect of the intervention relative to the control condition was analyzed using linear mixed-effects models. Compound outcome measures were standardized over time. Group (intervention versus control) was included as a factor. A random intercept was included in all models. Percentage of missingness of all outcome variables varied between 0.37 and 2.21%. *APOE* genotype information was missing for 5.89% of the participants. Missing values were not imputed as mixed-effects models adequately deal with missing data and multiple imputations provide no gain, regardless of the missing data mechanism [95]. Analyses were performed per protocol and according to the intention-to-treat (ITT) principle [96]. For the ITT analyses, participants were included if they had completed at least one measurement. Per-protocol analyses included only those participants who completed the intervention and all three measurements. For each outcome variable, we estimated the overall effect of the intervention after 9 months.

The linear mixed model analyses were repeated using the increase in physical activity between T0 and T2 as grouping variable, i.e., ≥ 35%, increase in average number of steps per day versus no increase, or < 35% in average number of steps per day at T2, 9 months after the start of the intervention. All analyses were adjusted for baseline performance, age, and sex (model A) and additionally for education level and *APOE* genotype (model B). Interactions between *APOE*, sex, and group on primary outcomes were investigated using interaction terms. Stratified analyses should be regarded as exploratory. Effect sizes (Cohen’s *d*) were calculated by dividing the regression coefficient by the pooled standard deviation of the outcome variable at baseline [97]. Significance level for all analyses was *0.0*5. Data were analyzed using IBM SPSS Statistics version 24 [98] and Stata version 16 [99].

## Results

### Numbers analyzed

Data collection was discontinued because of the COVID-19 outbreak. At this time, all participants had finalized the 6-month intervention. Eight participants (intervention *n* = 7, control *n* = 1) could no longer participate in the measurement 9 months after the start of the intervention (T2). At T2, data on the primary outcomes were available for 54/69 (78*.*26%) participants in the intervention group and 27/33 (81*.*82%) controls. There were no significant differences in baseline characteristics between the randomized participants who discontinued the intervention and those who completed the intervention. On average, the randomized participants who discontinued the intervention were slightly older than those who completed the intervention (*M* = 73.75, *SD* = 10.02; *M* = 69.90, *SD* = 8.81; *p* = 0.09). Figure 1 reviews the participant inclusion, withdrawals, and missing data. Serum concentrations were assessed at T0 and T1 only. At T0, serum was available for 64/69 (92*.*75%) participants in the intervention group and 30/33 (90*.*90%) participants in the control group. At T1, serum was available for 50/69 (72*.*46%) participants in the intervention group and for 26/33 (78*.*79%) controls.

### Baseline characteristics

Baseline characteristics are provided in Table 2. The mean age of the total sample was 70*.*69 years (SD = 9*.*16), and most participants were female (75*.*49%). The average age in the controls was 73*.*3 years (SD = 9*.*01) which is significantly higher than in the intervention group 69*.*45 years (SD = 9*.*02), *p* = *0.0*5.

**Table 2:** Baseline characteristics of the participants of the intervention stratified by randomization

Characteristics	Total sample (*n* = 102)	Intervention group (*n* = 69)	Control group (*n* = 33)	*P* value
Age	70.69 (9.16)	69.45 (9.02)	73.30 (9.01)	.05
Female	77 (75.49%)	50 (72.46%)	27 (81.82%)	.30
ApoE-ε4 status	29 (28.43%)	23 (33.33%)	6 (18.18%)	.11
Middle education	34 (33.33%)	23 (33.33%)	11(33.33%)	.99
High education	65 (63.73%)	43 (62.32%)	22 (66.67%)	.67
BMI	27.44 (4.37)	27.30 (4.30)	27.72 (4.57)	.65
Systolic blood pressure	142.62 (18.08)	141.63 (18.83)	144.68 (16.49)	.43
Waist Hip ratio	0.90 (0.08)	0.90 (0.09)	0.89 (0.08)	.48
**Medication**				
Statins	17 (16.67%)	9 (13.04%)	8 (24.24%)	.17
Anti-hypertensives	12 (11.76%)	8 (11.59%)	4 (12.12%)	.94
Antidiabetics	6 (5.88%)	3 (4.35%)	3 (9.09%)	.34
**Cognition**				
MMSE	29.35 (0.99)	29.35 (1.04)	29.36 (0.90)	.94
**Mobility**				
TUG	7.68 (2.96)	7.52 (2.44)	8.01 (3.86)	.43

Means and standard deviations, number and percentage reported. *P* values are based on univariate analyses of variance. *ApoE-ε4* Apolipoprotein E ε4 carrier, *BMI* Body mass index, *MMSE* Mini-Mental State Examination, *TUG* Timed Up and Go Test

### Intention to treat analyses

#### Primary outcomes

The ITT analysis (Table 3) shows a significant improvement in the average number of steps per day in the intervention group (*B* = 589*.*58; *p* = *.*047; *d* = 0*.*45) compared to the control group. However, the intervention has no significant effect on self-reported physical activity, physical fitness, or cognition. The correlation between the average number of steps and self-reported physical activity across all participants was low (*r* = 0.24). The intervention decreases balance. However, this difference is explained by an improvement in balance in controls over time (*B* =  − 0*.*76 [95% CI − 1*.*47 to − 0*.*05]; *p* = *.*04; *d* = 0*.*18). No effect moderation by ApoE-ε4 was observed for the primary outcomes.

**Table 3:** Results of the mixed models’ intention to treat analyses on primary outcomes including measures of physical activity, physical fitness, and cognition

**Outcome**	**Intervention group** ***N* = 69**	**Control group** ***N* = 33**	**Model A** ***N* = 102**			**Model B** ***N* = 96**		
**Physical activity**	**Mean (sd)**	**Mean (sd)**	**B (95% CI)**	***P* value**	***d***	**B (95% CI)**	***P* value**	***d***
**Steps per day**								
Baseline	4741.20 (1329.93)	4465.22 (1252.26)						
9 months	6708.67 (2971.22)	5595.89 (2594.14)	441.57 (− 107.69 to 990.82)	.12	.34	589.58 (8.78–1170.40)	**.047**	**.45**
**Self-reported PA**								
Baseline	113.00 (69.25)	108.67 (70.98)						
9 months	126.09 (70.67)	117.15 (71.04)	3.30 (− 11.52 to 18.12)	.66	.05	1.73 (− 14.14 to 17.60)	.83	.02
**Physical fitness**	**Mean (sd)**	**Mean (sd)**	**B (95% CI)**	***P* value**	***d***	**B (95% CI)**	***P* value**	***d***
**Aerobic capacity**								
Baseline	455.25 (87.49)	401.87 (78.92)						
9 months	488.68 (109.54)	437.92 (90.14)	− 2.31 (− 20.79 to 16.17)	.81	.03	− 0.63 (− 19.82 to 18.55)	.95	.01
**Muscle strength**^a^								
Baseline	− 0.12 (1.33)	− 0.69 (2.30)						
9 months	0.25 (1.44)	− 0.41 (1.95)	0.03 (− 0.15 to 0.21)	.78	.02	0.00 (− 0.19 to 0.20)	.96	0
**Balance**								
Baseline	38.52 (3.02)	37.06 (5.92)						
9 months	38.52 (3.18)	39.20 (2.77)	− 0.68 (− 1.33 to − 0.02)	**.04**	.16	− 0.76 (− 1.47 to − 0.05)	**.04**	**.18**
**Gait**								
Baseline	4.28 (1.17)	4.12 (1.02)						
9 months	3.90 (0.81)	4.28 (1.19)	− 0.14 (− 0.34 to 0.06)	.17^b^	.12	− 0.12 (− 0.33 to 0.09)	.25^b^	.11
**Cognition**	**Mean (sd)**	**Mean (sd)**	**B (95% CI)**	***P* value**	***d***	**B (95% CI)**	***P* value**	***d***
**Global cognition**^a^								
Baseline	− 0.02 (3.21)	− 0.63 (2.53)						
9 months	1.12 (3.58)	0.42 (3.02)	0.05 (− 0.31 to 0.42)	.77	.02	0.06 (− 0.31 to 0.44)	.76	.02
**Executive functioning**^a^								
Baseline	0.15 (2.44)	− 0.29 (2.30)						
9 months	0.69 (2.53)	0.24 (2.44)	0.16 (− 0.20 to 0.53)	.37	.07	0.20 (− 0.19 to 0.59)	.32	.08
**Learning**^a^								
Baseline	− 0.17 (1.67)	− 0.59 (1.06)						
9 months	0.27 (1.82)	0.41 (1.78)	− 0.20 (− 0.48 to 0.08)	.16	.11	− 0.16 (− 0.46 to 0.14)	.29	.11
**Verbal memory**^a^								
Baseline	− 0.21 (1.02)	− 0.18 (0.77)						
9 months	0.27 (1.06)	0.16 (0.85)	0.04 (− 0.15 to 0.23)	.69	.04	0.05 (− 0.16 to 0.25)	.66	.05

^a^Values standardized over time. ^b^Effect moderation by sex observed. *PA* Physical activity. Model A: adjusted for y at baseline, age, and sex. Model B: adjusted for y at baseline, age, sex, education, and ApoE-ε4 carrier status, *d* = Cohen’s *d*

**Fig. 3:**
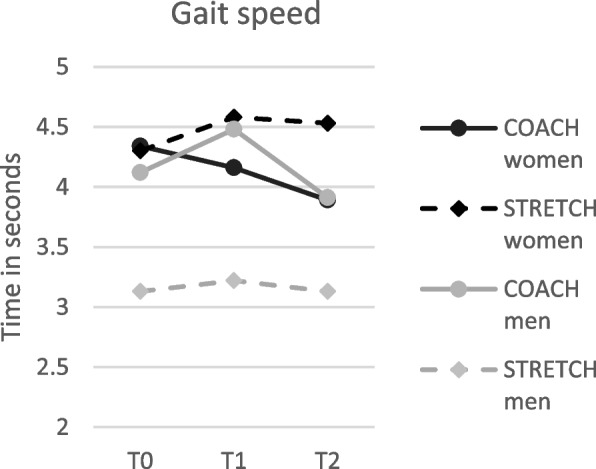
Average gait speed expressed in time in seconds needed to walk a 6-meter distance stratified by group and sex. Higher scores indicate a slower gait speed

The effect of the intervention on gait speed is significantly moderated by sex (*B* =  − 0*.*82 [95% CI − 1*.*30 to − 0*.*33]; *p* = *.*001). At baseline, the average time in seconds to walk 6 m is higher for women (M = 4.33, SD = 1.04) than for men (M = 3.93, SD = 1.33, *p* = 0.03), which indicates a slower gait speed in women. The women in the intervention group show an overall decrease in time in seconds, indicating an increase in gait speed. This increase in gait speed is significantly relative to the control group (*B* =  − 0*.*82 [95% CI − 1*.*30 to − 0*.*33]; *p* = *.*04;﻿ *d* = 0*.*73). The men in the intervention group show a slight overall increase in time in seconds. This increase occurs at T1, followed by a decrease at T2. See Fig. 3. This overall increase in time in seconds is not significant compared to the control group (*B* = 0.53 [95% CI − 0.01 to 1.07]; *p* = *.*0﻿6; *d* = 0*.*47).

#### Secondary outcomes—indicators of physical and mental health

The indices of frailty and depressive symptoms were unaffected by the intervention. The intervention decreased the number of ADL limitations (*B* =  − 0*.*11, *p* = *.*0﻿3; *d* = 0*.*30). The mental health score increased over time in the control group, indicating improved mental health, and remained constant in the intervention group (*B* =  − 2*.*48, *p* = *.*0﻿3, *d* = 0*.*21) (Table 4).

**Table 4:** Results of the mixed models’ intention to treat analyses on secondary outcome measures of physical and mental health

	**Intervention group** ***N* = 69**	**Control group** ***N* = 33**	**Model A** ***N* = 102**			**Model B** **N = 96**		
	**Mean (sd)**	**Mean (sd)**	**B (95% CI)**	***P* value**	***d***	**B (95% CI)**	***P* value**	***d***
**ADL limitations**								
Baseline	0.23 (0.86)	0.33 (0.69)						
9 months	0.23 (0.73)	0.42 (0.71)	− 0.10 (− 0.19 to − 0.004)	**.04**	**.12**	− 0.11 (− 0.21 to − 0.01)	**.03**	**.14**
**Experienced frailty**								
Baseline	0.11 (0.07)	0.12 (0.07)						
9 months	0.11 (0.06)	0.11 (0.07)	0.00 (− 0.01 to 0.01)	.46	0	0.00 (− 0.00 to 0.01)	.33	0
**Mental health**								
Baseline	81.67 (11.00)	79.96 (13.94)						
9 months	81.07 (12.85)	83.52 (11.61)	− 2.50 (− 4.51 to − 0.50)	**.02**	**.21**	− 2.48 (− 4.62 to − 0.34)	**.02**	**.21**
	**Intervention group** **N = 39**	**Control group** ***N* = 23**	**Model A** ***N* = 62**			**Model B** ***N* = 56**		
	**Mean (sd)**	**Mean (sd)**	**B (95% CI)**	***P* value**	***d***	**B (95% CI)**	***P* value**	***d***
**Depressive symptoms**								
Baseline	8.92 (6.05)	8.35 (6.67)						
9 months	7.64 (5.72)	8.30 (7.35)	− 0.67 (− 1.95 to 0.60)	.30	.11	− 0.86 (− 2.27 to 0.55)	.23	.14

Model A: adjusted for y at baseline, age, and sex. Model B: adjusted for y at baseline, age, sex, education, and ApoE-ε4 carrier status. *PA* Physical activity as measured in daily number of steps taken, *ADL* Activities of daily living, *IGF-I* Insulin-like growth factor 1, *HDL* High-density lipoprotein, *d* Cohen’s *d*

#### Secondary outcomes—serum concentrations assessed at 6 months (T1)

Serum concentrations of biomarkers of diabetes (insulin, IGF-I) and lipid metabolism (total cholesterol, HDL-cholesterol, and triglycerides) assessed at T1 were not significantly affected by the intervention (Table 5).

**Table 5:** Results of the mixed models’ intention to treat analyses serum concentrations 6 months after the start of the intervention (T1)

**Outcome**	**Intervention group** ***N* = 64**	**Control group** ***N* = 30**	**Model A** ***N* = 94**			**Model B** ***N* = 87**		
**Serum concentrations**	**Mean (sd)**	**Mean (sd)**	**B (95% CI)**	***P* value**	***d***	**B (95% CI)**	***P* value**	***d***
**IGF-I**								
Baseline	13.71 (4.48)	14.16 (4.37)						
6 months	14.58 (4.97)	14.47 (4.15)	0.23 (− 0.27 to 0.74)	.36	.05	0.21 (− 0.33 to 0.76)	.45	.05
**Insulin**								
Baseline	49.91 (33.74)	54.62 (37.49)						
6 months	43.55 (36.30)	46.18 (21.79)	0.88 (− 3.50 to 5.26)	.69	.03	1.44 (− 3.29 to 6.20)	.55	.04
**Total cholesterol**								
Baseline	5.34 (1.17)	5.29 (1.11)						
6 months	5.40 (1.17)	5.33 (1.02)	0.02 (− 0.08 to 0.12)	.70	.02	0.02 (− 0.09 to 0.13)	.69	.02
**HDL-cholesterol**								
Baseline	1.43 (0.44)	1.43 (0.36)						
6 months	1.45 (0.45)	1.48 (0.36)	− 0.01 (− 0.05 to 0.02)	.46	.02	− 0.01 (− 0.05 to 0.03)	.62	.02
**Triglycerides**								
Baseline	1.52 (1.04)	1.62 (0.94)						
6 months	1.51 (0.88)	1.55 (0.85)	0.02 (− 0.08 to 0.12)	.67	.02	0.02 (− 0.09 to 0.13)	.68	.02

Model A: adjusted for y at baseline, age, and sex. Model B: adjusted for *y* at baseline, age, sex, education, and ApoE-ε4 carrier status. *PA* Physical activity as measured in daily number of steps taken, *ADL* Activities of daily living, *IGF-I* Insulin-like growth factor 1, *HDL* High-density lipoprotein, *d* Cohen’s *d*

### Per-protocol analyses

The relative increase in the average number of steps per day in the intervention group did not reach significance in the per-protocol analyses (*B* = 679*.*16, *p* = *.*06). No significant intervention effects were observed for self-reported physical activity, physical fitness, and cognition. Significant moderation of the intervention effect by *APOE* was observed for learning and verbal memory. Stratification by genotype showed mixed results. In the controls, a relative improvement in performance on the learning index is observed only in those with ApoE-ε4 (*B* =  − 0*.*90, *p* = *.*0﻿1, *d* = 0*.*59) (Table 9, Appendix). Similar to the ITT analyses, significant effect moderation by sex was observed for the measure of gait speed. The results of the secondary outcomes correspond to those of the ITT analyses (Tables 10 and 11, Appendix). A description of the per-protocol analyses can be found in the Appendix.

### Responder analyses of those who achieved a 35% increase in daily physical activity

As a significant number of people in the intervention group did not reach the intended improvement in physical activity of 35%, we performed additional analyses comparing participants who achieved an increase in physical activity of  ≥ 35% (*N* = 35), to those who showed no increase in physical activity or less than 35% (*N* = 45). Those who achieved the intended improvement in physical activity of at least 35% were on average younger (*M* = 67.87, *SD* = 8.06; *M* = 71.67, *SD* = 9.09; *p* = .05) and had a higher level of cognitive functioning at baseline (Global cognition score *M* = 1.03, *SD* = 2.75; *M* =  − 0.87, *SD* = 3.04, *p* = .01) than those who did not achieve a 35% improvement in physical activity.

When comparing responders to non-responders, analyses show significant improvements in physical fitness and cognition. An increase in physical activity of  ≥ 35% over 9 months resulted in an improvement of aerobic capacity (*B* = 24*.*59, *p* = *.*02﻿, *d* = 0*.*26) and gait speed (*B* =  − 0*.*29, *p* = *.*02, *d* = 0*.*24). Balance, muscle strength, and self-reported level of physical activity were unaffected by the 35% increase in physical activity (*B* =  − 0*.*45, *P* value = *.﻿*23; *B* = 0*.*18, *p* = *.﻿*11 and *B* = 2*.*70, *p* = *.*77; *B* = 2.70, *p* = .77, respectively). In addition, we find improvements in global cognition (*B* = 0*.*65, *p* = *.﻿*002, *d* = 0*.*23), executive functioning (*B* = 0*.*72, *p* < *.﻿*001, *d* = 0*.*30), and verbal memory (*B* = 0*.*23, *p* = *.﻿*03, *d* = 0*.*23) compared to those that showed no increase or an increase in physical activity of less than 35%, in the fully adjusted models. Learning was unaffected by an increase in physical activity (*B* = 0*.*17, *p* = *.﻿*27) (Table 6).

**Table 6:** Results of the mixed models analyses investigating the effect of a >= 35% increase in physical activity on primary outcome measures including physical activity, physical fitness, and cognition

**Outcome**	** > = 35% increase PA T2** ***N* = 35**	** < 35% increase PA T2** ***N* = 45**	**Model A** ***N* = 80**			**Model B** ***N* = 75**		
**Physical activity**	**Mean (sd)**	**Mean (sd)**	**B (95% CI)**	***P* value**	***d***	**B (95% CI)**	***P* value**	***d***
**Steps per day**								
Baseline	4877.68 (1146.94)	4928.47 (1148.09)						
9 months	9204.67 (2303.47)	5312.41 (1836.38)	2002.22 (1515.78–2488.65)	**.000**	**1.76**	2079.88 (1560.74–2599.03)	**.000**	**1.82**
**Self-reported PA**								
Baseline	135.14 (70.88)	101.93 (68.45)						
9 months	145.31 (81.18)	111.51 (60.59)	1.59 (− 15.08 to 18.25)	.85	.02	2.70 (− 15.35 to 20.74)	.77	.03
**Physical fitness**	**Mean (sd)**	**Mean (sd)**	**B (95% CI)**	***P* value**	***d***	**B (95% CI)**	***P* value**	
**Aerobic capacity**								
Baseline	464.37 (75.46)	430.78 (102.97)						
9 months	527.74 (96.52)	456.61 (114.91)	21.40 (1.51–41.29)	**.04**	**.23**	24.59 (3.83–45.35)	**.02**	**.26**
**Muscle strength**								
Baseline	0.04 (1.32)	− 0.61 (2.11)						
9 months	0.55 (1.36)	− 0.23 (1.93)	0.13 (− 0.07 to 0.34)	.20	.07	0.18 (− 0.04 to 0.40)	.11	.10
**Balance**								
Baseline	39.04 (2.86)	37.38 (5.03)						
9 months	38.87 (3.37)	38.88 (2.93)	− 0.41 (− 1.08 to 0.26)	.23	.10	− 0.45 (− 1.18 to 0.28)	.23	.11
**Gait**								
Baseline	3.92 (0.85)	4.44(1.36)						
9 months	3.67 (1.04)	4.16 (0.88)	− 0.27 (− 0.48 to − 0.05)	**.02**	**.23**	− 0.29 (− 0.52 to − 0.10)	**.02**	**.24**
**Cognition**	**Mean (sd)**	**Mean (sd)**	**B (95% CI)**	***P* value**	***d***	**B (95% CI)**	***P* value**	***d***
**Global cognition**^a^								
Baseline	1.03 (2.75)	− 0.87 (3.04)						
9 months	2.91 (2.53)	0.12 (3.66)	0.52 (0.13–0.91)	**.009**	**.17**	0.65 (0.23–1.04)	**.002**	**.21**
**Executive functioning**^a^								
Baseline	0.71 (2.10)	− 0.30 (2.51)						
9 months	1.73 (2.07)	− 0.05 (2.58)	0.59 (0.22–0.96)	**.001**	**.25**	0.72 (0.33–1.11)	**.000**^**b**^	**.30**
**Learning**^a^								
Baseline	0.03 (1.56)	1.01 (1.37)						
9 months	− 0.30 (1.55)	0.24 (2.08)	0.17 (− 0.13 to 0.48)	.27	.11	0.17 (− 0.16 to 0.50)	.32	.11
**Verbal memory**^a^								
Baseline	0.01 (1.02)	− 0.35 (0.99)						
9 months	0.75 (0.83)	0.09 (1.00)	0.21 (0.01–0.40)	**.04**	**.21**	0.23 (0.02–0.44)	**.03**	**.23**

^a^Values standardized over time. ^b^Effect moderation by ApoE-ε4 observed. *PA* physical activity. Model A: adjusted for *y* at baseline, age, and sex. Model B: adjusted for *y* at baseline, age, sex, education, and ApoE-ε4 carrier status. *d* Cohen’s *d*

#### Secondary outcomes—indicators of mental and physical health

An increase in physical activity of  ≥ 35% at 9 months decreased the number ADL limitations (*B* =  − 0*.*13, *p* = *.﻿*02, *d* = 0*.*24). No differences were observed in experienced frailty (*B* =  − 0*.*01, *p* = *.*18), mental health (*B* = 0*.*79, *p* = *.﻿*55), or depressive symptoms (*B* =  − 0*.*86, *p* = *.﻿*28) (Table 7).

**Table 7:** Results of the mixed models analyses investigating the effect of a >= 35% increase in physical activity on secondary outcome measures of physical and mental health

	** > = 35% increase PA T2** ***N* = 35**	** < 35% increase PA T2** ***N* = 45**	**Model A** ***N* = 80**			**Model B** ***N* = 75**		
	**Mean (sd)**	**Mean (sd)**	**B (95% CI)**	***P* value**	***d***	**B (95% CI)**	***P* value**	***d***
**ADL limitations**								
Baseline	0.17 (0.45)	0.22 (0.60)						
9 months	0.14 (0.36)	0.33 (0.67)	− 0.08 (− 0.19 to 0.02)	.12	.15	− 0.13 (− 0.24 to − 0.02)	**.02**	**.24**
**Experienced frailty**								
Baseline	0.10 (0.05)	0.12 (0.07)						
9 months	0.10 (0.05)	0.12 (0.07)	− 0.01 (− 0.02 to 0.003)	.15	.16	− 0.01 (− 0.02 to 0.003)	.18	^.^16
**Mental health**								
Baseline	82.81 (10.73)	79.53 (13.84)						
9 months	83.99 (10.12)	82.81 (10.73)	0.79 (− 1.59 to 3.17)	.52	.06	0.79 (− 1.78 to 3.37)	.55	.06
	** > = 35% increase PA T2** ***N* = 26**	** < 35% increase PA T2** ***N* = 29**	**Model A** ***N* = 55**			**Model B** ***N* = 50**		
	**Mean (sd)**	**Mean (sd)**	**B (95% CI)**	***P* value**	***d***	**B (95% CI)**	***P* value**	***d***
**Depressive symptoms**								
Baseline	7.04 (5.29)	9.86 (6.95)						
9 months	6.08 (4.83)	9.00 (7.33)	− 0.36 (− 1.76 to 1.04)	.61	.06	− 0.86 (− 2.40 to 0.68)	.28	.14

Model A: adjusted for *y* at baseline, age, and sex. Model B: adjusted for *y* at baseline, age, sex, education, and ApoE-ε4 carrier status. *PA* Physical activity as measured in daily number of steps taken, *ADL* Activities of daily living, *IGF-I* Insulin-like growth factor 1, *HDL* High-density lipoprotein, *d* Cohen’s *d*

#### Secondary outcomes—serum concentrations assessed after 6 months (T1)

At 6 months, serum concentrations of biomarkers of diabetes (insulin, IGF-I) and lipid metabolism (total cholesterol, HDL-cholesterol, and triglycerides) were not significantly affected by the increase in physical activity (Table 8).

**Table 8:** Results of the mixed models analyses investigating the effect of a >= 35% increase in physical activity on serum concentrations 6 months after the start of the intervention (T1)

**Outcome**	** > = 35% increase PA T2** ***N* = 35**	** < 35% increase PA T2** ***N* = 38**	**Model A** ***N* = 71**			**Model B** ***N* = 71**		
**Serum concentrations**	**Mean (sd)**	**Mean (sd)**	**B (95% CI)**	***P* value**	***d***	**B (95% CI)**	***P* value**	***d***
**IGF-I**								
Baseline	14.55 (3.79)	13.87 (4.43)						
6 months	15.27 (3.80)	14.55 (3.79)	− 0.25 (− 0.85 to 0.35)	.41^a^	.06	− 0.31 (− 0.98 to 0.34)	.35^a^	.07
**Insulin**								
Baseline	53.84 (39.21)	51.93 (36.92)						
6 months	46.36 (46.01)	44.89 (22.09)	1.66 (− 3.74 to 7.05)	.55	.04	1.57 (− 4.33 to 7.48)	.60	.04
**Total cholesterol**								
Baseline	5.47 (1.25)	5.19 (1.15)						
6 months	5.58 (1.12)	5.18 (1.08)	0.02 (− 0.10 to 0.14)	.72	.02	0.03 (− 0.10 to 0.16)	.65	.03
**HDL-cholesterol**								
Baseline	1.41 (0.44)	1.45 (0.39)						
6 months	1.50 (0.44)	1.45 (0.39)	0.02 (− 0.02 to 0.06)	.38	.05	0.02 (− 0.02 to 0.07)	.34	.05
**Triglycerides**								
Baseline	1.43 (0.66)	1.62 (1.25)						
6 months	1.40 (0.74)	1.63 (0.96)	0.001 (− 0.13 to 0.13)	.99	0	− 0.02 (− 0.16 to 0.12)	.76	.02

^a^Effect moderation by sex observed. Model A: adjusted for *y* at baseline, age, and sex. Model B: adjusted for *y* at baseline, age, sex, education, and ApoE-ε4 carrier status

*PA* Physical activity as measured in daily number of steps taken, *ADL* Activities of daily living, *IGF-I* Insulin-like growth factor 1, *HDL* High-density lipoprotein, *d* Cohen’s *d*

#### Effect moderation by APOE

**Fig. 4:**
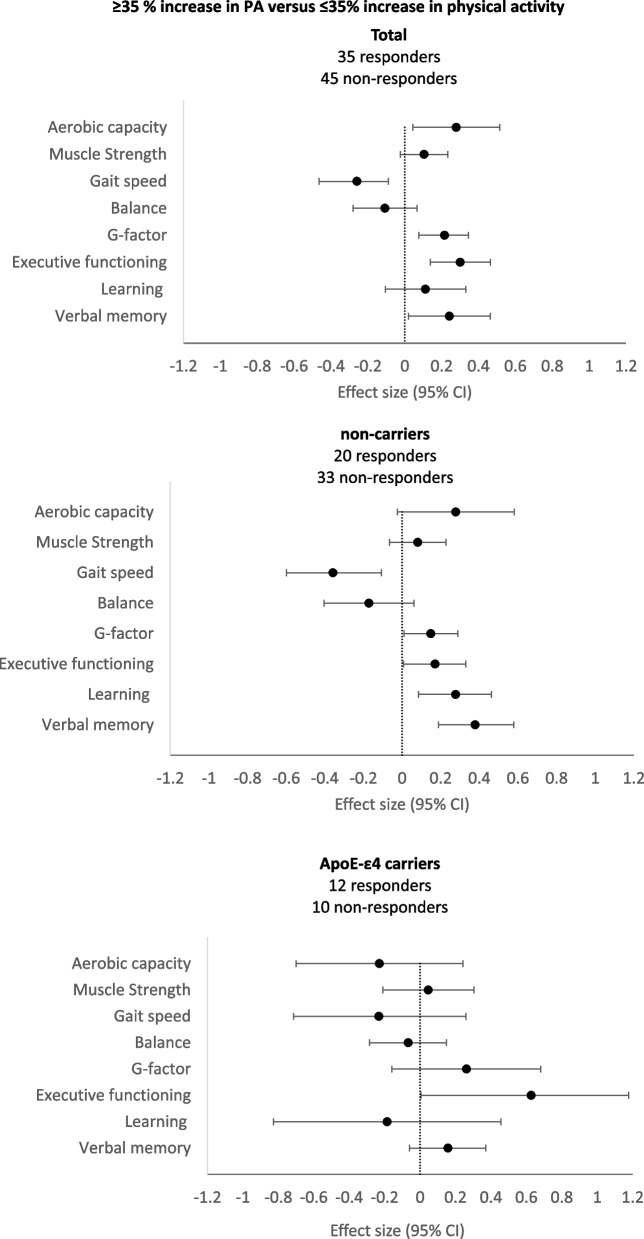
Forest plot of overall effect sizes of those who showed an increase in physical activity of at least 35% versus those who showed no increase in daily physical activity or an increase that was lower than 35% of their original level of physical activity at 9 months, stratified by genotype. Effect sizes are represented by Cohen’s *d*

The significant interaction between the 35% increase in physical activity and *APOE* on executive functioning (*B* = 1*.*22, *p* = *.*003, *d* = 0.51) indicates that increasing physical activity with ≥ 35% leads to greater improvement in executive functioning for those with ApoE-ε4 (*B* = 1*.*50, *p* = *.*003, *d* = 0*.*63) than for non-carriers (*B* = 0*.*41, *p* = *.﻿*04, *d* = 0*.*17). No significant effect moderation by *APOE* was observed for global cognition, learning, and verbal memory. Stratification by genotype shows significant effects of a ≥ 35% increase in physical activity on gait speed, global cognition, learning, and verbal memory only in ApoE-ε4 non-carriers and shows a large variance in the smaller group of ApoE-ε4 carriers. Figure 4 shows the effects of a ≥ 35% increase in physical activity, stratified by ApoE-ε4. Of note is that overall improvements in cognitive functioning were more robust and consistent in non-carriers.

## Discussion

The ITT analyses of primary outcomes show that the COACH method, aimed at the promotion of low-to-moderate physical activity, increases the level of physical activity of healthy elderly. No significant effects of the intervention are seen on self-reported physical activity, physical fitness, and cognition. Although the indices of self-reported physical activity, physical fitness, and cognition in the intervention group improve over time, a similar trend is seen in the controls. Stratification by sex shows opposing effects of the intervention in gait speed for men (lower gait speed) and women (higher gait speed) relative to controls. The secondary outcomes show a decrease in the number of ADL limitations in the intervention group and a relative improvement in the mental health of those in the control group. The intervention did not affect serum levels or measures of frailty or depression.

Additional responder analyses, focusing on the group that achieved the intended exposure of an increase in physical activity of  ≥ 35%, reveal that this group showed significant improvements in aerobic capacity and gait speed as well as verbal memory, executive functioning, and global cognition, compared to those who did not. The responder group did not show any significant improvements in the measures of balance, muscle strength, learning, frailty, mental health, serum levels, or depressive symptoms.

The null results of the ITT analyses of the intervention effect on cognition are at odds with the results from several meta-analyses that indicate that physical activity interventions have modest positive effects on cognition [6–10]. However, these trials show considerable heterogeneity in intervention type, outcome measures, and population attributes, which has lead several authors to conclude the experimental evidence is inconsistent or insufficient [11–14]. We hypothesize that the overall intensity of the promoted physical activity behaviors in the intervention group (low-to-moderate) in the present study was not high enough to achieve positive effects on physical fitness or cognition, relative to the active control group. Moderator analyses indicate that intensity and training duration are important effect moderators across studies, and the most successful interventions include the promotion of aerobic exercise of at least moderate intensity [7, 8, 11, 12, 15, 16, 100, 101].

The improvement in mental health observed in the control condition cannot be attributed to group differences in mental health score at baseline and might therefore be speculatively interpreted as a beneficial effect of the stretching exercises. A review of previous trials investigating the effects of yoga, a combination between stretching exercises and regulation of respiration, on mental health, has reported similar improvements in perceived stress and mental health [102]. However, this hypothesis remains to be confirmed in an independent study. While physical activity has been found to improve mental health in multiple studies, we do not see evidence for that in the intervention group.

The positive effects of the responder analyses on physical fitness and cognition suggest a threshold effect and substantiate the role of intensity and duration of physical activity behavior as important moderators of success. Based on the current findings, it remains unclear whether the effects of moderate-intensity aerobic exercise could also be achieved by equal energy expenditure during prolonged low-to-moderate physical activity. In addition, studies with a control condition with no contact or lifestyle education only more often resulted in significant intervention effects, whereas the presence of an active control or social group led to positive but no longer significant effects of the intervention, compared to controls [8].

The significant increase in average number of steps per day following the intervention was not reflected in a significant change in the self-reported physical activity score. Previous comparisons have found moderate correlations between pedometer assessments and self-reported activity (*r* = 0.33) [71]. In the present study, we found that both steps and self-reported physical activity increased after the intervention, but the correlation between both measures was lower than expected based on the literature (*r* = 0.24). Self-reported energy expenditure has been shown to be more strongly associated with psychological variables, like depression, attitudes toward physical activity, self-efficacy, and locus of control, than with health and anthropometric measures that predict engagement in physical activity [70]. In addition, the use of self-report questionnaires is more sensitive to response and recall bias, compared to more direct means of measurement. Self-report measures of physical activity might, therefore, also reflect more stable participant characteristics that are less likely to change as a consequence of a physical activity intervention.

Compared to controls and their male counterparts, the women in the intervention group show significantly more gain in average gait speed. This sex difference in the effect of the intervention on gait speed was not reported previously in the literature. Our finding could have been the result of regression to the mean or a higher compliance with the intervention in women. However, none of the other primary outcome measures suggested a difference in compliance between men and women. The relevance of this finding remains to be confirmed.

Looking at effect modification by *APOE*, the effects of an increase in physical activity of  ≥ 35% on cognition are most robust and consistent in ApoE-ε4 non-carriers. However, a significant group-by-genotype interaction indicates that a ≥ 35% increase in physical activity leads to greater improvement in executive functioning for ApoE-ε4 carriers. This finding is in line with the idea that protective associations between physical activity and brain health might be more pronounced in ApoE-ε4 carriers than in non-carriers [32, 37, 38, 40, 103–107].

The increase in physical activity following the intervention did not lead to an increase in the level of circulating IGF-I or to a significant improvement in the biomarkers of the cardiovascular risk profile, the number of depressive symptoms, and levels of experienced frailty and mental health as expected based on the literature [108–123]. We hypothesize that the power, duration, and intensity of the present study were insufficient to detect the expected protective effects. The assessment of the serum biomarkers, in particular, might have been too early in the intervention to detect any effects. Observational data from epidemiological studies indicate that moderate physical activity, when sufficiently sustained, can slow the age-related progression of frailty and depressive symptoms [110–114, 122] and mitigation of cardiovascular risk factors and diabetes mellitus largely depends on sustained regular physical activity throughout the lifespan [124].

The strength of our trial is the accessibility of the intervention for those vulnerable to accelerated cognitive decline. Dropout rates were under 25%. Comprehensive measurement led to the detection of modest changes in a wide range of functions. The effects of an improvement in physical activity of  ≥ 35% on cognition are small (Cohen’s *d* 0*.*17–0*.*30) but span a wide range of highly relevant cognitive functions.

## Limitations

The present trial has some limitations. First, the number of participants in the control group that completed the study was lower than indicated in the power calculations. As a consequence, the power might have been insufficient to detect the expected effects and effect estimates might be biased. Stratified analyses should be regarded as exploratory. Second, the number of participants in the intervention group that achieved the intended improvement in physical activity was too low, suggesting the intervention was feasible but not effective enough to establish a significant change in physical activity in most of the participants. Third, converting non-ambulatory activities to steps, according to the criteria of Tudor-Locke and Basset [64, 65, 125], might have introduced subjectivity. Fourth, the duration of pedometer use was not standardized across groups. The difference in the length of the registration period might have led to an overestimation of the levels of physical activity in the control group, due to reactivity in the first week of measurement [67]. Lastly, because of the long pre-dementia stage of neuropathological changes, we cannot fully exclude bias by reverse causation, in that those with the highest level of cognitive functioning might profit more from exercise counseling, than those with lower levels of cognitive functioning at baseline.

## Conclusion

In summary, although not all participants achieved the intended improvement in physical activity, the tailored intervention was easily accessible and well appreciated in a group of elderly with low levels of physical activity. The ITT analyses did not show intervention effects on physical fitness or cognition. Responder analyses suggest benefits on physical fitness and cognition for those who managed to achieve an increase in physical activity ≥ 35% over 9 months. In light of the potential benefits of physical activity on physical fitness and cognitive functioning, we highly recommend an ambitious approach towards the prevention of cognitive decline and dementia by stimulating sustained engagement in moderate to high levels of physical activity, over the course of the lifetime. Future studies could optimize intervention potential by aiming at a sustained increase in physical activity of  ≥ 35% in a population of “at risk” individuals defined by various health parameters including age, level of physical activity, and genetic susceptibility. This study also reveals several difficulties in physical activity trials, which are difficult to blind for participants and researchers. Intervention potential could be further enhanced by replacing the active control condition with a no-contact or waitlist control condition and further standardization of the assessment of physical activity by means of actigraphy or accelerometry.

## Data Availability

All data collected for the study are under embargo until 2024, unless permission is granted by the principal investigator ES. After the expiration of the embargo access to the deidentified participant data, informed consent, study protocol, and a data dictionary defining each field can be requested via the principal investigator. Access to the data will be granted after approval of the research proposal, with a signed data access agreement.

## Appendix

### Per-protocol analyses

#### Primary outcomes

The per-protocol analyses showed no significant effects of the intervention on the average number of steps per day (*B* = 679.16 [95% CI − 25.99 to 1384.31]; *p* = .06).

No effect of the intervention on self-reported physical activity or measures of physical fitness and cognition was observed (Table 9). Significant effect moderation by sex was observed for the measure of gait speed (*B* =  − 0.84 [95% CI − 1.42 to − 0.27]; *p* = .004). Stratification by sex showed neither men nor women in the intervention group experienced significant changes in gait speed, relative to controls. Significant effect moderation by *APOE* genotype was observed for measures of verbal memory (*B* =  − 0.60 [95% CI − 1.14 to − 0.06]; *p* = .03) and learning (*B* =  − 0.89 [95% CI − 1.71 to − 0.08]; *p* = .03). Stratification by genotype showed a greater improvement in learning in the control group in ApoE-ε4 carriers only (*B* =  − 0.90 [95% CI − 1.62 to − 0.18]; *p* = .01, *d* = 0.59). Stratification did not yield significant intervention effects on verbal memory in either group.

**Table 9:** Results of the mixed models per-protocol analyses on primary outcomes including measures of physical activity, physical fitness, and cognition

**Outcome**	**Intervention group** ***N***** = 54**	**Control group** ***N***** = 27**	**Model A** ***N***** = 81**			**Model B** ***N***** = 76**		
**Physical activity**	**Mean (sd)**	**Mean (sd)**	**B (95% CI)**	***P***** value**	***d***	**B (95% CI)**	***P***** value**	***d***
**Steps per day**								
Baseline	4995.66 (1165.82)	4661.67 (1106.72)						
9 months	7634.80 (2688.64)	5799.16 (2688.64)	516.79 (− 133.60 to 1167.17)	.12	.45	679.16 (− 25.99 to 1384.31)	.06	.59
**Self-reported PA**								
Baseline	115.56 (70.49)	116.07 (73.07)						
9 months	127.56 (70.59)	122.56 (74.57)	− 1.57 (− 19.33 to 16.18)	.86	.02	− 6.27 (− 25.39 to 12.85)	.52	.09
**Physical fitness**	**Mean (sd)**	**Mean (sd)**	**B (95% CI)**	***P***** value**	***d***	**B (95% CI)**	***P***** value**	***d***
**Aerobic capacity**								
Baseline	470.57 (88.00)	396.31 (81.67)						
9 months	509.72 (113.27)	439.76 (95.95)	− 10.10 (− 32.90 to 12.69)	.39	.11	− 12.38 (− 36.44 to 11.68)	.31	.13
**Muscle strength**^a^								
Baseline	− 0.04 (1.35)	− 0.89 (2.43)						
9 months	0.43 (1.47)	− 0.54 (2.04)	0.03 (− 0.19 to 0.25)	.78	.02	0.01 (− 0.23 to 0.25)	.92	.01
**Balance**								
Baseline	38.77 (2.56)	36.85 (6.31)						
9 months	38.79 (3.14)	39.09 (3.04)	− 0.60 (− 1.31 to 0.11)	.10	.14	− 0.69 (− 1.48 to 0.10)	.09	.16
**Gait**								
Baseline	4.24 (1.24)	4.14 (1.07)						
9 months	3.76 (0.73)	4.34 (1.26)	− 0.18 (− 0.42 to 0.06)	.14^b^	.15	− 0.15 (− 0.41 to 0.11)	.25^b^	.13
**Cognition**	**Mean (sd)**	**Mean (sd)**	**B (95% CI)**	***P***** value**	***d***	**B (95% CI)**	***P***** value**	***d***
**Global cognition**^a^								
Baseline	0.34 (3.28)	− 0.70 (2.43)						
9 months	1.73 (3.59)	0.53 (3.11)	0.02 (− 0.41 to 0.46)	.91	.01	− 0.02 (− 0.48 to 0.45)	.94	.01
**EF**^a^								
Baseline	0.37 (2.46)	− 0.27 (2.17)						
9 months	0.99 (2.52)	0.26 (2.45)	0.19 (− 0.23 to 0.60)	.37	.08	0.19 (− 0.27 to 0.65)	.41	.08
**Verbal memory**^a^								
Baseline	− 0.19 (1.09)	− 0.19 (0.83)						
9 months	0.43 (1.04)	0.23 (0.90)	0.05 (− 0.17 to 0.27)	.68	.05	0.05 (− 0.19 to 0.29)	.69^**c**^	.05
**Learning**^a^								
Baseline	0.07 (1.70)	− 0.58 (1.09)						
9 months	0.55 (1.81)	0.61 (1.88)	− 0.28 (− 0.61 to 0.05)	.10	.18	− 0.27 (− 0.65 to 0.09)	.14^**c**^	.17

^a^Values standardized over time. ^b^Effect moderation by sex observed. ^c^Effect moderation by *APOE ε4* observed. Model A: adjusted for *y* at baseline, age, and sex. Model B: adjusted for *y* at baseline, age, sex, education, and ApoE-ε*4* carrier status, *d* Cohen’s *d*

#### Secondary outcomes—indicators of physical and mental health

The per-protocol analyses showed an effect of the intervention on the number of experienced ADL limitations (*B* =  − 0.13, [95% CI − 0.26 to − 0.01], *p* = .03, *d* = 0.35). The TOPICS-MDS mental health score increases over time in the control group and remains constant in the intervention group (*B* =  − 2.73, [95% CI − 5.46 to − 0.002], *p* = .05, *d* = 0.22) (Table 10).

**Table 10:** Results of the mixed models per-protocol analyses on secondary outcomes—indicators of physical and mental health

	**Intervention group** ***N***** = 54**	**Control group** ***N***** = 27**	**Model A** ***N***** = 81**			**Model B** ***N***** = 76**		
	**Mean (sd)**	**Mean (sd)**	**B (95% CI)**	***P***** value**	***d***	**B (95% CI)**	***P***** value**	***d***
**ADL limitations**								
Baseline	0.11 (0.37)	0.37 (0.74)						
9 months	0.15 (0.41)	0.44 (0.75)	− 0.12 (− 0.23 to − 0.003)	**.045**	.**22**	− 0.13 (− 0.26 to − 0.01)	**.03**	**.24**
**Experienced frailty**								
Baseline	0.11 (0.05)	0.12 (0.08)						
9 months	0.11 (0.06)	0.11 (0.08)	0.01 (− 0.01 to 0.02)	.37	.16	0.01 (− 0.01 to 0.02)	.27	.16
**Mental health**								
Baseline	81.77 (11.14)	79.47 (15.06)						
9 months	81.28 (13.73)	84.00 (12.40)	− 2.89 (− 5.41 to − 0.37)	**.03**	**.23**	− 2.73 (− 5.46 to − 0.002)	**.05**	**.22**
	**Intervention group** ***N***** = 35**	**Control group** ***N***** = 20**	**Model A** ***N***** = 55**			**Model B** ***N***** = 50**		
	**Mean (sd)**	**Mean (sd)**	**B (95% CI)**	***P***** value**	**d**	**B (95% CI)**	***P***** value**	***d***
**Depressive symptoms**								
Baseline	8.66 (6.07)	8.30 (6.90)						
9 months	7.23 (5.62)	8.30 (7.66)	− 0.74 (− 2.15 to 0.68)	.31	.12	− 0.89 (− 2.42 to 0.63)	.25	.14

Model A: adjusted for *y* at baseline, age, and sex. Model B: adjusted for *y* at baseline, age, sex, education, and *APOE ε4* carrier status. *PA* Physical activity as measured in daily number of steps taken, *ADL* Activities of daily living, *IGF-1* Insulin-like growth factor 1, *HDL* High-density lipoprotein, *d* Cohen’s *d*

#### Secondary outcomes—serum concentrations assessed at 6 months (T1)

Serum concentrations of biomarkers of diabetes (insulin, IGF-1) and lipid metabolism (total cholesterol, HDL-cholesterol, and triglycerides) assessed at 6 months were not significantly affected by the intervention (Table 11).

**Table 11:** Results of the mixed models per-protocol analyses on serum concentrations assessed at 6 months (T1)

**Outcome**	**Intervention group** ***N***** = 50**	**Control group** ***N***** = 25**	**Model A** ***N***** = 75**			**Model B** ***N***** = 70**		
**Serum concentrations**	**Mean (sd)**	**Mean (sd)**	**B (95% CI)**	***P***** value**	***d***	**B (95% CI)**	***P***** value**	***d***
**IGF-1**								
Baseline	14.32 (4.04)	13.82 (4.34)						
6 months	15.80 (3.77)	14.58 (4.10)	0.42 (− 0.24 to 1.08)	.21	.10	0.41 (− 0.33 to 1.15)	.28	.10
**Insulin**								
Baseline	50.37 (36.57)	56.79 (39.78)						
6 months	43.64 (40.51)	48.10 (21.24)	0.82 (− 5.15 to 6.79)	.79	.02	2.25 (− 4.39 to 8.88)	.51	.06
**Total cholesterol**								
Baseline	5.40 (1.21)	5.21 (1.17)						
6 months	5.51 (1.21)	5.16 (0.93)	0.01 (− 0.12 to 0.14)	.91	.01	0.002 (− 0.15 to 0.15)	.98	.002
**HDL-cholesterol**								
Baseline	1.42 (0.46)	1.48 (0.37)						
6 months	1.47 (0.47)	1.51 (0.33)	− 0.03 (− 0.08 to 0.01)	.14	.07	− 0.03 (− 0.08 to 0.02)	.18	.07
**Triglycerides**								
Baseline	1.51 (0.76)	1.54 (1.14)						
6 months	1.57 (0.96)	1.41 (0.63)	0.06 (− 0.07 to 0.20)	.37	.06	0.06 (− 0.09 to 0.22)	.42	.06

Model A: adjusted for y at baseline, age, and sex. Model B: adjusted for y at baseline, age, sex, education, and *APOE ε4* carrier status. *PA* Physical activity as measured in daily number of steps taken, *ADL* Activities of daily living, *IGF-1* Insulin-like growth factor 1, *HDL* High-density lipoprotein, *d* Cohen’s *d*

## Abbreviations

15 WT: 15 Word test
ADL: Activities of daily living
*APOE*: Apolipoprotein gene
ApoE-ε4: Apolipoprotein E ε4 allele
BDNF: Brain-derived neurotropic factor
BMI: Body mass index
CI: Confidence interval
CES-D: Centre for Epidemiological Studies depression scale
DNA: Deoxyribonucleic acid
HDL: High-density lipoprotein
IGF-I: Insulin-like growth factor I
ITT: Intention to treat
LLT: Location learning test
MMSE: Mini-Mental Status Examination
PA: Physical activity as measured in daily number of steps taken
PARQ: Physical Activity Readiness Questionnaire
PASE: Physical activity scale for the elderly
SPPB: Short physical performance test
SD: Standard deviation
TMT: Trail making test
TUG: Timed Up and Go Test
